# Hard and soft tissue regeneration of severe peri-implantitis defects with the laser-assisted peri-implant defect regeneration technique: 3-year results

**DOI:** 10.1186/s40729-023-00467-1

**Published:** 2023-02-05

**Authors:** Robert Noelken, Laura Westphal, Eik Schiegnitz, Bilal Al-Nawas

**Affiliations:** 1 Private Practice for Oral Surgery, Paradiesplatz 7-13, 88131 Lindau, Lake Constance Germany; 2 Department of Oral and Maxillofacial Surgery, University Medical Center, Johannes Gutenberg University of Mainz, Augustusplatz 2, 55131 Mainz, Germany; 3 Private Practice, Bad Homburg, Germany

**Keywords:** Dental implant, Peri-implantitis, Bone graft, Soft tissue graft, Reconstructive therapy, Bone regeneration, Recession, Keratinized mucosa, Surface decontamination

## Abstract

**Purpose:**

This retrospective cohort study evaluates the regeneration of severe peri-implantitis deficiencies treated with the laser-assisted peri-implant defect regeneration (LAPIDER) approach within a 3-year follow-up.

**Methods:**

Twenty-four implants with severe peri-implantitis in 18 patients were treated according to the LAPIDER technique. In contrast to classic techniques for reconstructive peri-implantitis surgery with a marginal incision, a buccal split-flap preparation avoiding papillae separation was used. After a coronal flap elevation and a laser-assisted peri-implant defect cleaning, connective tissue and autogenous bone grafting was performed. Primary outcomes were the changes of the marginal bone levels (MBL) and the buccal bone thickness. Secondary outcomes included implant survival, peri-implant probing depths (PPD), bleeding on probing (BOP), recession, width of keratinized mucosa (KMW), thickness of keratinized mucosa (KMT), soft tissue esthetics (PES), and implant success.

**Results:**

MBL improved interproximal by 3.10 ± 2.02 mm (*p* < 0.001), buccal by 3.49 ± 2.89 mm (*p* < 0.001), and lingual by 1.46 ± 1.98 mm (*p* = 0.003); buccal bone thickness by 0.55 ± 0.60 mm (*p* = 0.005), and 1.01 ± 1.25 mm (*p* = 0.001) at 1 and 3 mm below reference level. Two implants were removed; 22 implants were still in function at a mean follow-up of 36 months. PPD changed from 5.05 ± 1.39 to 3.08 ± 0.71 mm (*p* < 0.001); recession was reduced from 2.07 ± 1.70 to 0.91 ± 1.13 mm (*p* = 0.001); KMW increased from 2.91 ± 1.81 to 4.18 ± 1.67 mm (*p* = 0.006); KMT improved from 1.73 ± 0.50 to 2.44 ± 0.43 mm (*p* < 0.001); PES changed from 7.7 ± 2.8 to 10.7 ± 1.9 (*p* < 0.001). 45.8% to 54.2% of the implants met the criteria of implant success.

**Conclusions:**

The favorable results document the proof of principle for the regeneration of severe peri-implant hard and soft tissue deficiencies by the LAPIDER treatment approach.

## Introduction

The primary goal of peri-implantitis therapy is to resolve the peri-implant inflammation with a remission of the suppuration and BOP, to reduce the PPD, to stop additional loss of the supporting bone and to keep the implant in function. Studies have shown that a successful remission of the peri-implant infection is possible [1, 2]. The additional aims of regenerative peri-implantitis therapy are a re-osseointegration, a vertical bone gain, an improvement of KMW and KMT, a reduction of the recession and an improvement of the peri-implant PES.

A systematic review observed the efficacy of reconstructive surgical peri-implantitis therapy in 16 studies (3 with control groups), which reported their outcomes at 12 months after surgery. For reconstruction of the bony defect autologous, allogeneic or xenogeneic bone grafting, guided bone regeneration, and the application of biological agents and growth factors were included. A larger improvement in marginal bone levels of 1.7 mm (57% defect fill) was found in the groups grafted with bone graft material comparing to control groups. In all 16 studies the mean marginal bone level improved by 2.0 mm [3].

A recent randomized multi-center study evaluated the 1-year outcomes of surgical peri-implantitis therapy. In the test group, the peri-implant defects were grafted with a demineralized bovine bone mineral (DBBM) containing collagen (Bio-Oss Collagen®, Geistlich, Lucerne, Switzerland). With or without DBBM defect grafting the radiographic defect fill was 1.1 mm in sites with a marginal bone loss of 6.1 mm at baseline [4]. In another multi-center study with a similar set-up, DBBM (BioOss, Geistlich) covered by a collagen membrane (BioGide, Geistlich) was used in the test group [5]. In contrast to the previous study, they found significantly more radiographic defect fill in the test group of 2.3 mm (baseline 4.4 mm) in comparison to the control group of 1.1 mm (baseline 4.9 mm). Even when the treatments in the previous studies were successful, the mean PPD at 1-year examination were still between 4.5 and 4.9 mm, and the marginal bone levels between 2 and 5 mm below implant shoulder. Additionally, no differences were revealed for the clinical measurements (PPD, BOP) when compared to control groups, while all groups reported an increase of recession between 0.5 and 1.1 mm. In contrast, studies using autogenous bone in peri-implantitis therapy are rarely found, but report favorable potential of vertical bone regeneration between 3.0 and 4.7 mm [6, 7].

A major challenge of re-osseointegration of an infected implant is the successful elimination of the bacterial biofilms from the contaminated implant surface. Numerous tools for mechanical debridement of the implant surface are available without a clear superiority of one technique [8]. Various types of curettes, rotary titanium brushes [9], ultrasonic tips, and air powder abrasion are options for effective cleansing without severe damage the implant surface [10]. A new electrolytic approach presented no bacterial re-growth after in vitro treatment [11], and a significant radiographic defect fill of about 3 mm after 18 months [12]. The effectiveness of the Er:YAG laser was compared to hand instrumentation and chlorhexidine for nonsurgical treatment of peri-implantitis and it presented a statistically significant higher reduction of BOP [2]. The major advantage of the Er:YAG laser over all other techniques for surface decontamination is that this technique can be applied also without an open-flap procedure in a setting with limited access to the defect site.

Classic surgical concepts in the treatment of periodontal or peri-implantitis lesions most often use a marginal full-thickness access flap with separation of the papilla complex to access and clean the defect site. An incision above a bony lesion is contrary to basic surgical rules and diminishes the blood supply and regeneration capacity in the critical interproximal zone. This might be a reason that those techniques most often lead to a partial defect regeneration, a soft tissue volume reduction, and an increase in recession [13, 14]. Recently, a new technique for periodontal regenerative surgery with a buccal apical incision avoiding the marginal papilla separation was presented [15, 16]. Importantly, the combination of apical access flap and connective tissue grafting led to improved soft tissue outcomes with a reduction of PPD and recession.

**Fig. 1:**
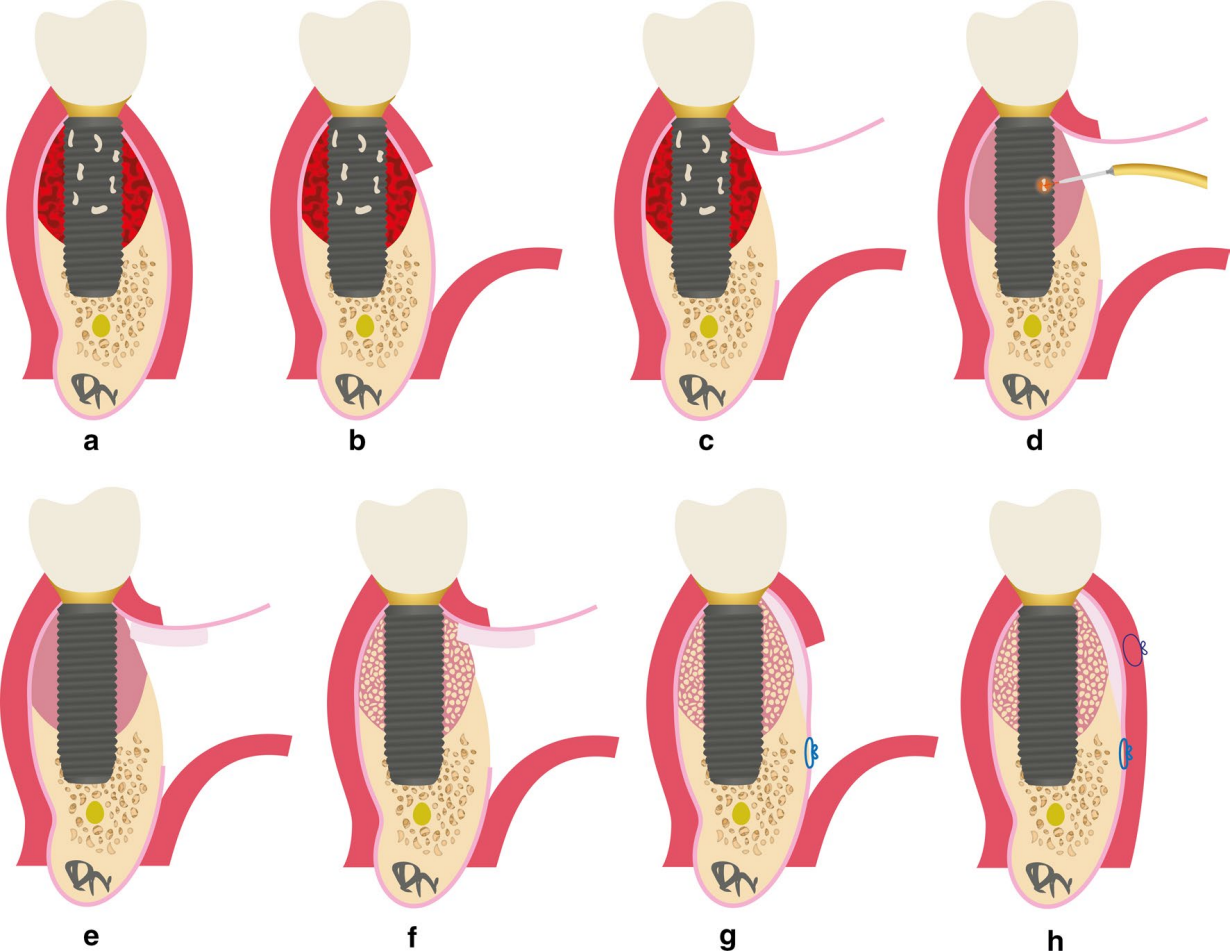
Illustration of the LAPIDER treatment approach. **a** Presence of a severe peri-implantitis defect. **b** Split-flap preparation from mucogingival border in apical direction. **c** Apical separation of the periosteum and subperiosteal coronal flap elevation. **d** Peri-implant defect cleaning and decontamination of the implant surface by the Er:YAG laser. **e** Subperiosteal connective tissue grafting. **f** Peri-implant defect augmentation with autogenous bone chips. **g** Periosteal mattress suturing with resorbable suturing material. **h** Mucosal suturing for a bilaminar wound closure

Today, patients with a severe peri-implant hard and soft tissue deficiency are not only looking for the remission of the infection, but they also desire additionally an esthetic improvement by an increase in healthy tissue volume. Therefore, we need new techniques which solve the peri-implant infection as well as enable a sufficient hard and soft tissue grafting with an increase of the soft tissue height and volume. To overcome these problems, a new surgical regenerative peri-implantitis treatment approach was developed. In contrast to classic concepts, the LAPIDER concept uses a buccal split-flap preparation without separation of the papillae. This enables the access and debridement of the bone defect, the decontamination of the implant surface with an Er:YAG laser, and the grafting with autogenous bone and connective tissue (Fig. 1) [17].

This retrospective cohort study evaluates the outcome of implants with severe peri-implantitis treated with the LAPIDER approach within a mean follow-up period of 3 years.

## Materials and methods

The primary outcome parameter of this study was the peri-implant marginal bone regeneration which was reflected by interproximal, buccal and lingual marginal bone level changes as well as the buccal bone thickness.

The secondary outcome parameters were the implant survival, peri-implant probing depths, bleeding on probing, midbuccal recession, width and thickness of the keratinized mucosa, peri-implant soft tissue esthetics, and implant success.

### Patients and implants

This retrospective cohort study included implants with severe peri-implantitis treated in the time range from September 2018 to June 2020.

Inclusion criteria were as follows: severe peri-implantitis with bleeding on probing, suppuration, and radiographically confirmed bone loss. Exclusion criteria were known or suspected current malignancy, history of radiation therapy in the head and neck region, chemotherapy within 5 years prior to surgery, uncontrolled diabetes mellitus, permanent immunosuppressive medication, bone modifier (e.g., bisphosphonate or rank ligand inhibitor) medication, and present alcohol and/or drug abuse. Smoking was not regarded as an exclusion criterion.

Twenty-four implants in 18 patients (11 females, 7 males) with a mean age of 54.1 ± 12.0 years were included. Fourteen patients were non-smokers while 4 were smokers (1 smoker with more than 15 cigarettes a day, 3 smoker with 6 to 10 cigarettes a day). Twelve patient showed a thick gingival biotype while 6 patients exhibited a thin gingival biotype. All 24 implants from various brands (2 Straumann Bone Level, 1 Straumann Tissue Level, 3 Ankylos, 2 Brånemark System, 2 NobelActive, 2 NobelPerfect, 1 Frialit I, 6 OsseoSpeed, 3 OsseoSpeed Profile, 1 Camlog Rootline, 1 ICX) were treated according to the new approach. Eight implants were treated in the posterior mandible, 14 in the anterior maxilla and 2 in the posterior maxilla.

### Ethical approval

Since no study-related additional radiographs or examinations were performed and the publication of the obtained data was planned anonymously, the Ethics Committee of the state Rhineland-Palatinate, Germany (file 2020-15366) decided that no votum was necessary for this cohort study. The study was conducted according to the recommendations of good clinical practice. Written informed consent was obtained from all patients prior to any examination carried out.

### Pre-treatment examination

At pre-treatment examination, subjects in need of a peri-implantitis treatment were screened for eligibility to the study. A periapical X-ray and often a CB-CT were recorded to evaluate the circumferential marginal bone levels at the implant site. The PPD, the BOP, the suppuration, the amount of recession, the PES and the KMW were also documented. The gingival biotype was assessed by a periodontal probe [18]. In case of an insufficient KMW, an apically positioned flap with free gingival graft (FGG) was performed prior to the regenerative peri-implantitis surgery.

### Surgical technique

Following the diagnosis of a severe peri-implantitis (Fig. 1a), all implants were treated according to the LAPIDER technique, which has been described previously [19]. As a first therapy step, a horizontal mucosal incision 4 to 5 mm apical to the marginal mucosa with a length of approximately 20 mm was created. A supraperiosteal split-flap was prepared in apical direction with the support of a scalpel and micro-scissors (Fig. 1b). At the level of the apex, the periosteum was separated horizontally, followed by a subperiosteal flap elevation in coronal direction to get access to the peri-implant lesion (Fig. 1c). After separation of the granulation tissue from the periosteum, the peri-implant defect was explored and debrided with hand instruments and curettes. Following the removal of the granulation tissue, the implant surface was cleaned and decontaminated with the Er:YAG laser (AdvErL EVO, PS600T tip, set to 50 mJ/mm^2^, 20 pps, J. Morita Corp. Europe, Dietzenbach, Germany) (Fig. 1d). The lingual aspect of the defect and contaminated implant surface was reached by the slim laser tip through the lingual pocket under optical control from the buccal aspect. These procedures were carried out using a chair-side microscope. A connective tissue graft was harvested at the palate and fixed subperiosteally mesial and distal of the bony lesion (Fig. 1e). For the augmentation of the bone defect, autogenous bone chips were harvested by a bone scraper (Micross, Meta, Reggio Emilia, Italy) in the mandibular ramus and stored in doxyxycline (100 mg in 5 ml injection solution) for at least one minute. The peri-implant defect was filled with the autogenous bone chips and condensed with a plugger (Fig. 1f). To cover the hard and soft grafts, the periosteum was reflected apically and sutured to the periosteum with resorbable suture material (Fig. 1g). For a bilayered wound closure, the mucosa was sutured on top of the periosteum to the mucosa (Fig. 1h). A 10-day antibiotic peri-operative prophylaxis (amoxicillin 2 × 750 mg plus metronidazole 2 × 500 mg daily) was administered starting 3 days prior to surgery. Rinsing with 0.2% chlorhexidine was prescribed for starting at the 5th post-surgical day.

### Follow-up and definition of outcome variables

**Fig. 2:**
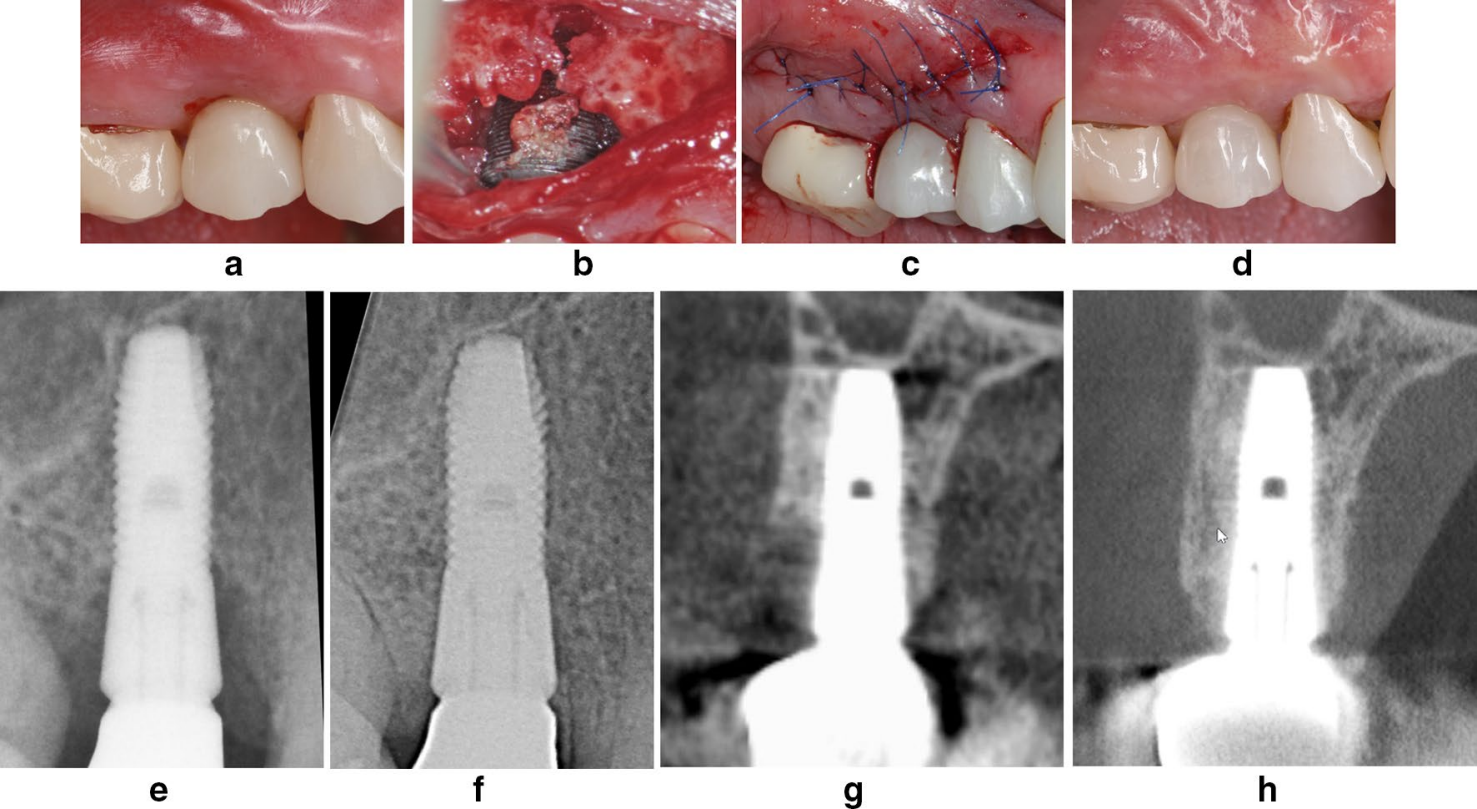
**a** Severe peri-implantitis lesion with suppuration and bleeding on probing. **b** Access to the severe buccal peri-implant bony lesion by the LAPIDER approach. **c** Hard and soft tissue defect grafting and bilaminar wound closure. **d** At 3-year follow-up examination no signs of a peri-implant infection are left. **e** The baseline radiograph reveals a severe interproximal bone loss. **f** A radiograph at 2-year follow-up examination shows the complete interproximal regeneration. **g** The pre-operative CB-CT shows a severe buccal bone loss. **h** At 3-year follow-up a CB-CT reveals the complete bone regeneration to the implant shoulder level

**Fig. 3:**
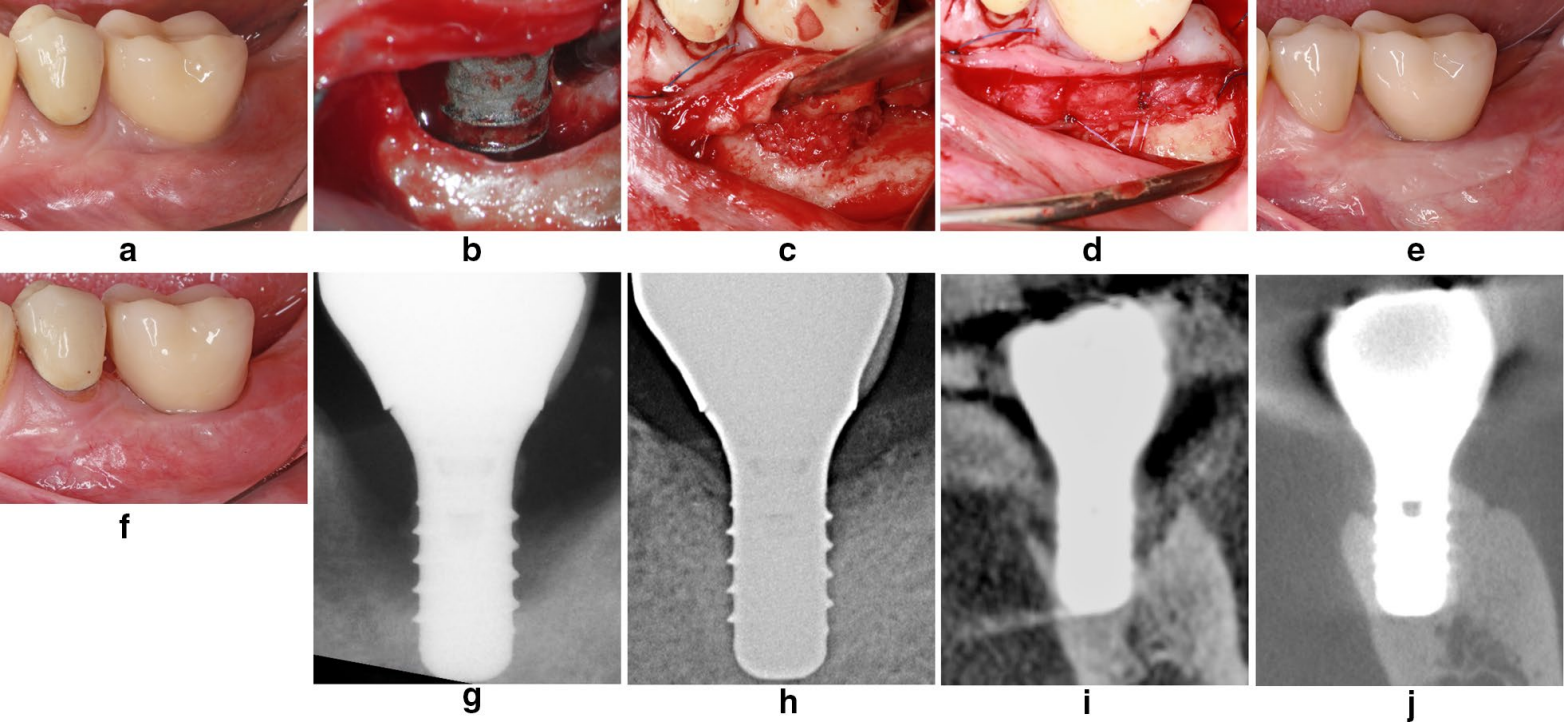
**a** Severe peri-implantitis lesion with suppuration and an insufficient width of the keratinized mucosa. **b** Implant surface decontamination with the Er:YAG laser. **c** Hard and soft tissue grafting according to the LAPIDER technique. **d** Periosteal suturing to cover the autogenous grafts. **e** Uneventful healing 9 months after LAPIDER surgery, but still without a sufficient zone of attached mucosa. **f** 3-year follow-up examination after an additional apically positioned flap with FGG. **g** Severe interproximal bone loss at pre-operative examination. **h** Radiograph at 3-year follow-up examination reveals significant vertical bone regeneration. **i** Severe buccal and lingual bone loss at baseline. **j** CB-CT at 3-year follow-up examination shows a pronounced buccal and lingual bone regeneration

The patients were examined preoperatively; at the time of peri-implantitis surgery; and at 1-, 2- and 3-year follow-up examinations (Figs. 2 and 3).

### Evaluation of primary outcome parameters

#### Interproximal marginal bone level

The status of the interproximal marginal bone level was assessed using digital periapical radiographs. Attachment levels crestal to a reference level were designated as positive values and vice versa. The interproximal bone level was calculated by a mean of the mesial and distal bone level.

#### Buccal and lingual bone level and buccal bone thickness

The pre- and post-operative status of the buccal and lingual bone wall was determined by CB-CT data and was specifically reconstructed according to the long axis of the implants. The thickness of the buccal bone wall was measured at 1, 3 and 6 mm apical to the reference level [20, 21], which was defined by the marginal level of the microstructure at the treated implant design. The increase in thickness of the buccal bone wall was calculated by the difference between the final and the pre-operative thickness of the buccal bone wall.

### Evaluation of secondary outcome parameters

#### Implant survival

Implant survival was estimated according to the Kaplan–Meier method [22].

#### Peri-implant probing depths

The PPDs were measured at 6 sites around the implant by a periodontal probe with 1 mm calibration.

#### Bleeding on probing

The presence or absence of BOP was recorded following the measurement of the peri-implant probing depths at 6 sites.

#### Soft tissue recession

The midbuccal recession was calculated in relation to a tangent between the cemento-enamel junctions of the neighboring teeth by a periodontal probe with 1 mm calibration.

#### Width of keratinized mucosa

The KMW at the midbuccal aspect of the implant sites was measured by a periodontal probe with 1 mm calibration.

#### Buccal mucosa thickness

The buccal mucosa thickness was evaluated by an ultrasonic device with 20 MHz frequency and a 1540 m/s ultrasonic impulse velocity (PIROP Biometric Scanner; Echoson, Pulawy, Poland). The KMT at a level 4 mm apical to the midfacial mucosal margin at the implant was measured. The duration of return from the echo of the ultrasonic impulse was determined and the distance was calculated and digitally displayed to the nearest 0.01 mm. Ten measurements were performed to calculate an arithmetic mean. Minimal pressure was applied to avoid compression of the keratinized mucosa.

#### Peri-implant soft tissue esthetics

The esthetics of the per-implant soft tissues was evaluated according to the PES established by Fürhauser [23].

#### Implant success

Three composite indexes of success were calculated according to the criteria established by Renvert [5] and Derks [4]:

**Composite index 1:** implant not lost, radiographic defect fill ≥ 1 mm, peri-implant probing depths ≥ 5 mm at all aspects, absence of bleeding on probing (1 out of 6 measurements at implant site accepted), and absence of suppuration at all aspects.
**Composite index 2:** implant not lost, radiographic defect fill ≥ 0 mm, PPD ≥ 5 mm at all aspects, no BOP, and no suppuration at any aspects.
**Composite index 3:** implant not lost, absence of BOP or suppuration at all aspects, PPD ≥ 5 mm at all aspects, and recession ≥ 1 mm at the buccal aspect of the implant.

### Statistical analysis

Survival probabilities were estimated by the Kaplan–Meier method for all implants. The analysis exploring the linkage between gain in marginal bone level and the PES improvement, and between the gain in marginal bone and the KMW at final examination, utilized the Spearman’s rank-based correlations. Subpopulations within the study group (smokers vs. non-smokers, thin vs. thick mucosal biotype, with or without additional apically positioned flap) were compared using the non-parametric Mann–Whitney *U*-test. The reported *p*-values are two-sided. Results were considered statistically significant at *p* ≥ 0.05. For graphic description, boxplots are given. All calculations were carried out using SPSS 22 (SPSS Inc., Chicago, USA).

## Results

### Primary outcome parameter

**Fig. 4:**
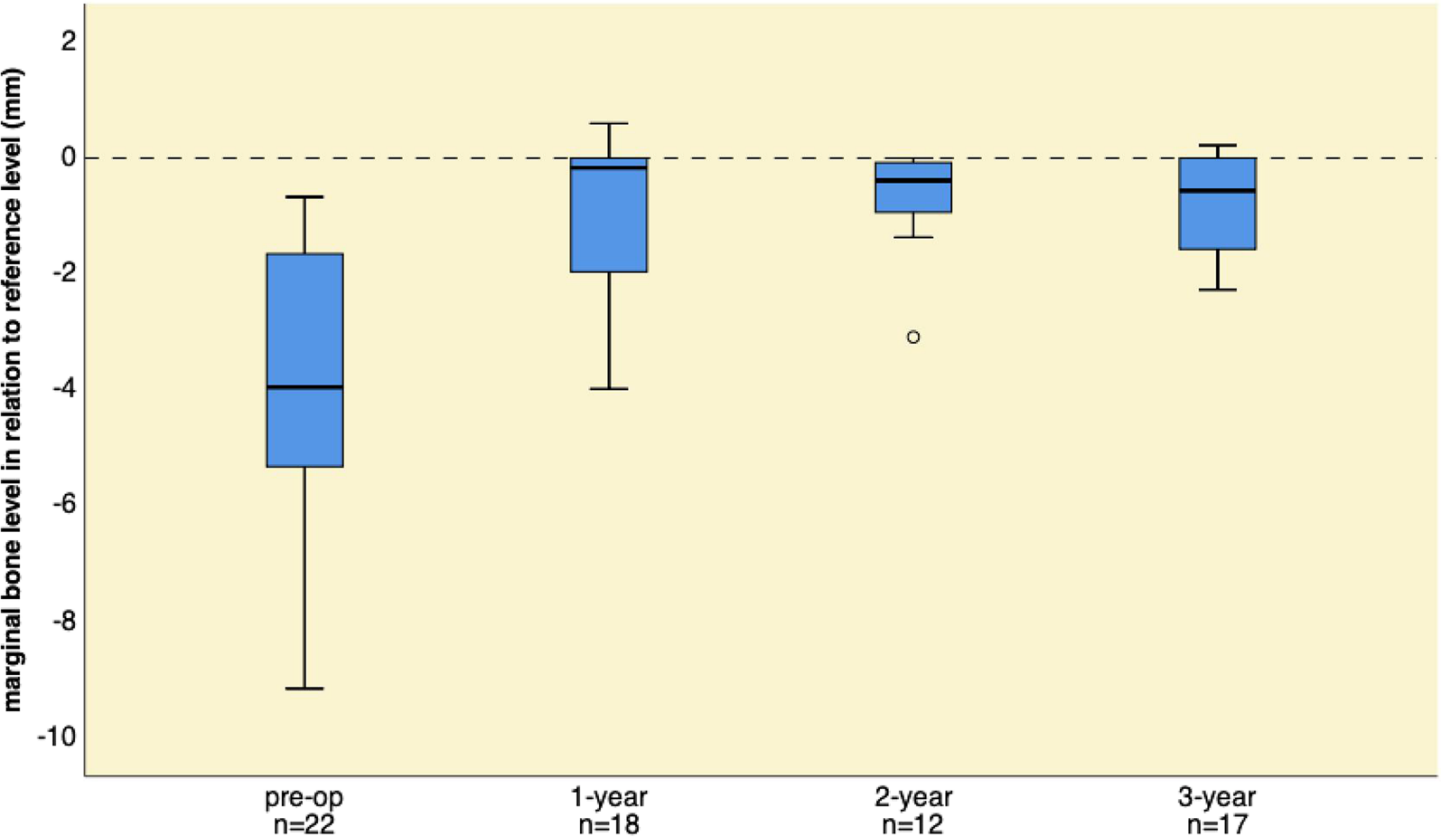
Interproximal marginal bone-level changes from pre-operative examination to 1-year, to 2-year, and to 3-year follow-up examinations

The interproximal marginal bone level changed from pre-operatively − 3.74 ± 2.27 mm (*n* = 22), to − 0.85 ± 1.31 mm at 1 year (*n* = 18), to − 0.68 ± 0.88 mm at 2 years (*n* = 12), and to − 0.81 ± 0.95 mm at 3 years (*n* = 17) (Fig. 4) in relation to the reference level. At the final examination, the interproximal MBL was − 0.64 ± 0.92 mm with a mean vertical bone gain of 3.10 ± 2.02 mm (*p* < 0.001).

The buccal MBL changed from pre-operatively − 4.64 ± 2.39 mm to − 1.15 ± 1.73 mm at the final examination. The mean vertical buccal bone gain was 3.49 ± 2.89 mm (*p* < 0.001).

The lingual MBL changed from pre-operatively − 2.24 ± 2.52 mm to − 0.78 ± 1.25 mm at the final follow-up with a mean vertical lingual gain of 1.46 ± 1.98 mm (*p* = 0.003).

The buccal bone thickness changed at the levels 1, 3 and 6 mm apical to the reference level from pre-operatively 0.01 ± 0.03 mm, 0.32 ± 0.57 mm, and 1.26 ± 1.24 mm to 0.55 ± 0.60 mm, 1.32 ± 1.16 mm, and 1.90 ± 1.52 mm at the final examination. The changes at 1 mm by 0.55 ± 0.60 mm (*p* = 0.005), and at 3 mm by 1.01 ± 1.25 mm (*p* = 0.001) reached the level of significance (Table 1).

**Table 1:** Changes of the thickness of the buccal bone wall at the level 1, 3, and 6 mm apical to reference level

Level	Pre-op (mm)	Final (mm)	Improvement (mm)	*p* =
1 mm	0.01 ± 0.03	0.55 ± 0.60	0.55 ± 0.60	0.005
3 mm	0.32 ± 0.57	1.32 ± 1.16	1.01 ± 1.25	0.001
6 mm	1.26 ± 1.24	1.90 ± 1.52	0.65 ± 1.73	0.163

### Secondary outcome parameters

#### Implant survival

**Fig. 5:**
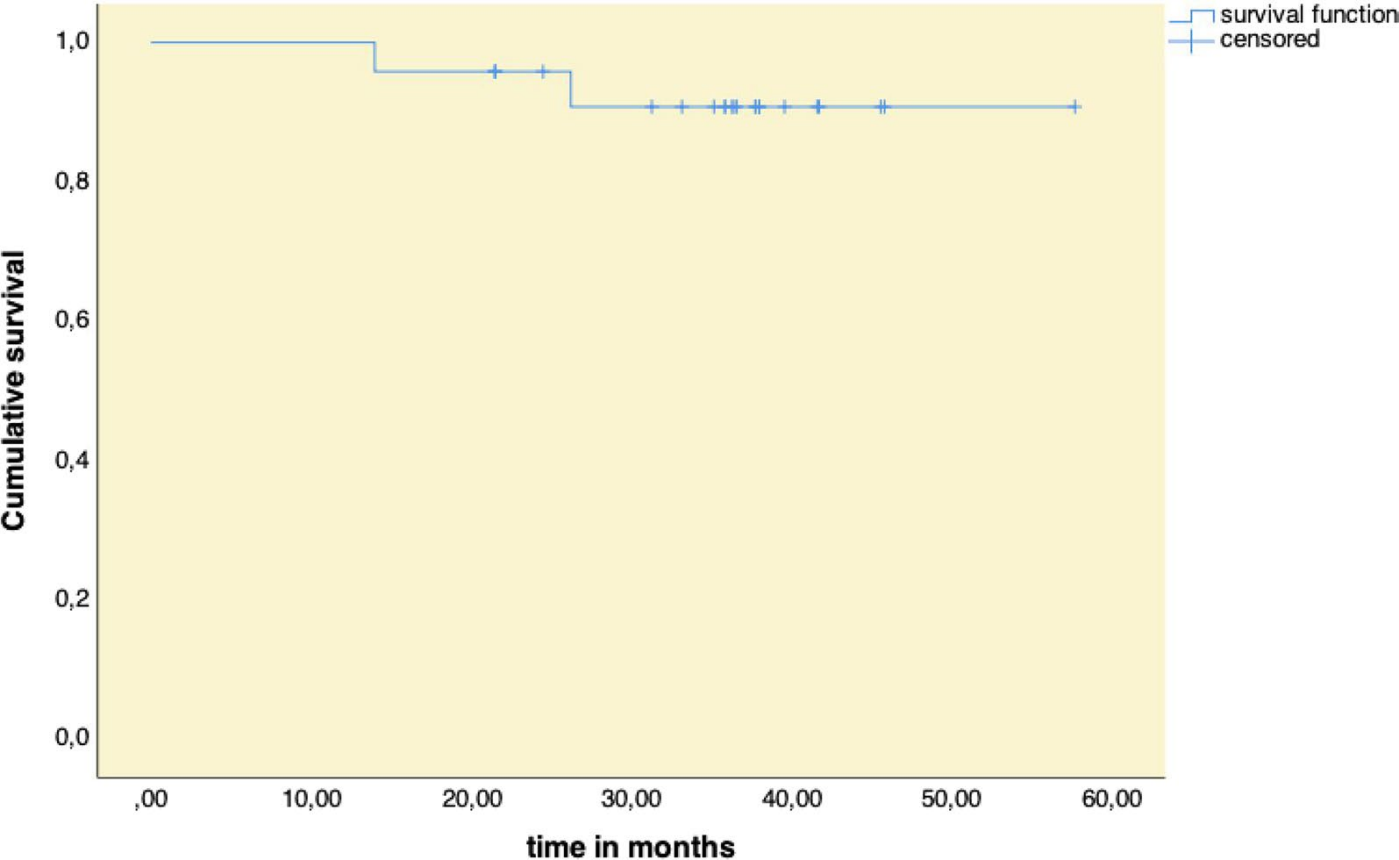
Survival function according to Kaplan–Meier for all 24 implants treated by the LAPIDER concept

Two implants were removed due to progressive PPD after 14 and 26 months, respectively. One of them was a 30-year-old experimental Frialit-1 implant with a threaded osseointegrated apical part and a 5 mm long parallel non-threaded coronal part which showed severe peri-implant bone loss and suppuration. Although this implant was treated according to the LAPIDER protocol, the implant showed progressive PPD with suppuration. The implant in position of a upper lateral incisor was removed without flap elevation and replaced immediately with a new implant with a simulteneous hard and soft tissue reconstruction in tunnel technique. Another implant (Straumann bone level) placed in the position of a second mandibular molar, presented no attached lingual peri-implant mucosa and did not show any improvement following a LAPIDER prodecure and an apically positioned flap with FGG regarding the lingual probing depths; this implant was removed without further replacement. The remaining 22 implants were still in function at a mean follow-up period of 36.0 ± 8.6 months (range, 21.5 to 57.7 months) without any signs of peri-implant infection or suppuration. The cumulative survival rate according to the Kaplan Meier methods was 90.8% (Fig. 5).

#### Peri-implant probing depths

The mean PPD changed significantly from pre-operatively 5.05 ± 1.39 mm to 3.08 ± 0.71 mm at final examination (*p* < 0.001). For details in probing depths see Table 2.

**Table 2:** Pre-operative and final PPD at 6 sites around the observed implants (in mm)

	Pre-op				Final			
	Mesial	Central	Distal	Mean	Mesial	Central	Distal	Mean
Buccal	5.16 ± 1.89	5.43 ± 2.12	5.23 ± 1.92	5.27 ± 1.75	2.98 ± 0.96	2.77 ± 0.81	3.41 ± 1.12	3.05 ± 0.71
Lingual	5.00 ± 1.73	4.50 ± 1.74	4.98 ± 1.33	4.83 ± 1.28	3.46 ± 1.05	2.73 ± 1.00	3.16 ± 1.25	3.11 ± 0.94

#### Bleeding on probing

The freqency of BOP after probing at 6 sites was 100% at the implant sites at pre-operative, and 36.4% at final examination (*p* < 0.001).

#### Midbuccal recession

The mean depth of buccal recession changed from 2.07 ± 1.70 to 0.91 ± 1.13 mm (*n* = 22; *p* = 0.001). The mean gain of the midbuccal soft tissue level was 1.16 ± 0.96 mm. No significant difference was found for sites with (1.05 ± 0.93 mm) or without an apically positioned flap (1.09 ± 1.16 mm) (*p* = 0.949).

#### Width of keratinized mucosa

The KMW at the midbuccal aspect of the implant site improved significantly from 2.91 ± 1.81 mm pre-operatively to 4.18 ± 1.67 mm at the final examination (*p* = 0.006). The mean gain of KMW was 1.27 ± 1.73 mm. In 11 sites an apically positioned flap was performed pre- (*n* = 2) or post-operative (*n* = 9) with (*n* = 7) or without (*n* = 4) an additional FGG to improve the KMW. The gain of KMW was 0.14 ± 1.07 mm in sites without additional apically positioned flap (*p* = 0.717), but 2.41 ± 1.51 mm in sites with additional apically positioned flap (*p* = 0.005).

#### The thickness of the keratinzed mucosa

The KMT increased from 1.73 ± 0.50 to 2.44 ± 0.43 mm (*p* < 0.001) by 0.71 ± 0.50 mm. No signifant difference was found in sites with (0.56 ± 0.31 mm) or without (0.85 ± 0.62 mm) an apically positioned flap (*p* = 0.270).

#### Peri-implant soft tissue esthetics

The mean pink esthetic score changed from 7.7 ± 2.8 pre-operatively to 10.7 ± 1.9 at the final follow-up examination (*p* < 0.001). The detailed pre- to 3-year post-operative changes of the variables of PES are displayed in Table 3. The most critical variables of the PES were the distal papilla and the buccal soft tissue level which both increased significantly within the 3-year observation period. At the 22 remaining implants sites an improved or stable score of the PES was noticed.

**Table 3:** Mean score (± SD) of the variables of the PES according to Fürhauser during the observation period

PES variables	Pre-op *n* = 22	1-year *n* = 17	2-year *n* = 12	3-year *n* = 17	Final *n* = 22	*p* = Pre-op to final
Papilla mesial	1.0 ± 0.8	1.3 ± 0.6	1.3 ± 0.7	1.2 ± 0.6	1.2 ± 0.5	0.059
Papilla distal	0.8 ± 0.6	1.2 ± 0.6	1.4 ± 0.5	1.1 ± 0.7	1.3 ± 0.7	0.002
Soft tissue level	1.0 ± 0.8	1.3 ± 0.7	1.4 ± 0.5	1.3 ± 0.8	1.3 ± 0.8	0.035
Soft tissue contour	1.1 ± 0.6	1.4 ± 0.5	1.3 ± 0.5	1.5 ± 0.5	1.4 ± 0.5	0.059
Alveolar process	1.1 ± 0.6	1.8 ± 0.4	1.8 ± 0.5	1.8 ± 0.4	1.8 ± 0.4	0.001
Soft tissue color	1.3 ± 0.5	1.8 ± 0.4	1.9 ± 0.3	1.8 ± 0.4	1.8 ± 0.4	0.002
Soft tissue texture	1.1 ± 0.6	1.9 ± 0.2	2.0 ± 0.0	1.9 ± 0.3	1.9 ± 0.3	< 0.001
Sum PES	7.7 ± 2.8	10.7 ± 2.2	11.2 ± 1.6	10.7 ± 2.0	10.7 ± 1.9	< 0.001
Median	8.0	11.0	11.0	11.0	10.5	
Range	3–13	6–14	6–14	7–14	7–14	

#### Implant success

With a mean follow-up of 36 months, the different implant success criteria were met in varying degrees. The majority of implants that survived (91.7%) presented without further bone loss (91.7%) and a marginal bone defect fill greater than 1 mm (87.5%); with shallow PPD smaller or equal than 5 mm (83.3%); without any buccal recession (≥ 1 mm; 100%); and no suppuration on probing (95.8%). In contrast, the complete absence of BOP was achieved only for 58.3% of the implants. According to the endpoint definition of the composite criteria 1, 2 and 3, the cumulative success rates were 45.8%, and 50%, and 54.2%, respectively.

#### Correlation results

No significant differences were found when comparing the amount of interproximal MBL gain in smoker vs. non-smokers (*p* = 0.810); in patients with thick vs. thin mucosal biotype (*p* = 0.955); or with or without an additional apically positioned flap (*p* = 0.867). No significant correlation between the gain in MBL and the improvement of the PES was found (*r* = − 0.172; *p* = 0.443) as well as between the gain in MBL and the KMW at thefinal examination (*r* = − 0.319; *p* = 0.147).

## Discussion

Using data from mean follow-up examinations of 36 months, this cohort study analyzed the peri-implant marginal bone and soft tissue changes as well as implant survival and success from using the new LAPIDER approach for the surgical therapy of severe peri-implantitis.

Within the observation period, favorable implant survival and success rates, significant improvements of the circumferential MBL, the buccal marginal bone thickness, the PES, the KMW and KMT were observed. Correspondingly, signs of infection (PPD, BOP) and the buccal recession decreased significantly. Radiographs and CB-CT data revealed a significant vertical bone regeneration and a thickening of the buccal bone wall even when the initial status of the circumferential bone levels was severely compromised.

In accordance with a treatment concept for severe periodontitis, this study implemented a perioperative prophylaxis whereby amoxicillin and metronidazole were administered [24]. In the non-augmentative as well in the regenerative therapies, the decontamination of the implant surface is the most critical part of the therapy [25]. Adjunctive application of systemic antibiotics is suggested in several studies. A 1- and 3-year follow-up study assessed the administration of amoxicillin to surgical peri-implantitis therapy [1]. Even at 1 year the cases with modified implant surfaces showed advantages; at 3 years no significantly better outcome was detectable anymore. The systematic review by Oen et al. critically evaluated the additional use of systemically administered antibiotics in the open surgical treatment of peri-implantitis [26]. The authors concluded that the use of systemically administered antibiotics as a supplement to surgical peri-implantitis therapy cannot be justified as a part of a standard treatment protocol. Systemic antibiotics in nonsurgical treatment of peri-implantitis shows a weak effect, whereas for surgical non-reconstructive treatment no effect is demonstrated [27]. This seems to be paradoxical since most studies on regenerative peri-implantitis surgery apply biomaterials and use a systemic perioperative antibiotic prophylaxis [4, 5].

A recently published placebo-controlled study evaluated outcomes after nonsurgical peri-implantitis therapy with or without adjunctive systemic metronidazole [28]. After 12 months, in the metronidazole group, a significantly greater PPD reduction (2.53 vs. 1.02 mm) and more radiographic bone gain (2.33 vs. 1.13 mm) were documented. Additionally, the number of pathogenic bacteria dropped significantly.

Finally, the prescription of antibiotics and the time of intake in peri-implantitis therapy must be considered critically in every case taking into account the severeness of infection, the aims of therapy as well as the risk of complications and resistances.

In implant sites with a total loss of the peri-implant attached mucosa at the baseline examination, an apically positioned flap with a FGG graft was performed prior to LAPIDER surgery in the present study. Additionally, in the post-operative phase an apically positioned flap was performed with or without an additional FGG when the zone of attached mucosa seemed to be too limited to conduct a sufficient oral homecare maintenance [29]. A recent review emphasized again the importance of a zone of keratinized and attached mucosa for peri-implant health [30]. Surgical techniques for the modification the peri-implant soft tissue stability were observed in a systematic review. An apically positioned flap with a FGG, a collagen membrane, or an acellular dermal matrix showed a significant increase in KMW compared to non-grafted sites. The gain in KMW was significantly higher in sites where the apically positioned flap was used in combination with a FGG compared to sites without FGG or any other material [31].

In this study a new surgical access to the peri-implant lesion was used to prevent the separation of the interproximal papilla complex. This allowed the cleaning and augmentation of the defect with hard and soft tissues as well as to cover the graft completely. An incision above a pathologic bony lesion, which is used in most classic periodontal and peri-implant surgical concepts, is contrary to basic surgical treatment rules and destroys the blood supply in a critical area for wound healing and may reduce the amount of bone regeneration. These ideas are based on the concept that incision lines should avoid the area directly above the graft and should be placed in the vestibular area (“poncho-flap”) [32].

A major challenge with an important impact on bone regeneration is the complete removal of bacterial biofilms from the implant surface [33].

Surface roughness and thread design of the implants increase osseointegration but are disadvantageous when they are exposed and in need of removal of the adhered plaque and biofilm. Numerous tools for mechanical and electrolytic debridement procedures of the implant surface (scalers, glycine powder, titanium rotary brush, electrolytic procedure, laser) are available and successful [9–11, 34–37]. Since the surgical access to the peri-implant lesion is more delicate with the presented LAPIDER technique, only a few instruments are possible to use with this approach which allows to leave the prosthetic restoration in place. In this cohort study an Er:YAG laser with a 0.6 mm diameter tip was used, which allows access also in limited situations the lingual bony defects via the lingual pocket under visual control from the buccal aspect.

In a clinical study, the effectiveness of an Er:YAG laser in comparison to that of mechanical debridement using plastic curettes and antiseptic therapy with chlorhexidine for nonsurgical treatment of peri-implantitis was observed. At 6 months after treatment, both therapies led to significant improvements of the investigated clinical parameters, but the Er:YAG laser group presented a statistically significant higher reduction of bleeding on probing [38]. In a recent case report, a new approach for the treatment of severe peri-implantitis was presented [39]. With the use of the Er:YAG laser, the granulation tissue, the calcified deposit and the biofilm were removed effectively from the implant surface during surgery.

In the present study, autogenous bone chips harvested from the mandibular ramus were used for grafting of the peri-implant deficiency. These were stored before application in doxycycline for at least 1 min.

Although autologous bone often is referred to as the “gold standard”, studies using autologous bone are rarely found. In an older prospective study with 25 implants, the treatment of peri-implantitis defects using autogenous particulated and block bone grafts was evaluated [6]. The median marginal bone loss was reduced from 6.2 to 2.3 mm. In an animal model, autogenous bone grafts alone or in combination with a polytetrafluoroethylene membrane, a membrane alone or a conventional flap procedure, were compared. A mean bone gain of 4.7 mm was identified around implants treated with bone and membrane, 4 mm at sites with autogenous bone alone, 3 mm at sites with a membrane alone and 1.9 mm at the control sites [7]. Tetracyclines have been observed extensively for their antibiotic activity, anti-inflammatory effect, and inhibition of collagenolytic enzymes responsible for the degradation of connective tissue and bone. In an animal study using albino rats, the bone repair of critical-size defects with the support of doxycycline (a semisynthetic derivative of tetracycline) was evaluated [40]. The results demonstrate that the combination of 10% doxycycline with autogenous bone showed a higher bone formation than a blood control group, doxycycline alone, or autogenous bone alone.

In contrast to autogenous bone, the peri-implant defect augmentation with DBBM is well documented in literature. Bovine-derived is the most frequently used xenograft in several clinical studies for grafting of peri-implantitis defects. In a randomized, multi-center clinical trial, the surgical treatment of peri-implantitis with full-thickness flap, surface cleaning using rotating and titanium brushes, and augmentation with or without DBBM and a collagen membrane was followed for 12 months. The radiographic defect fill followed by grafting with DBBM (2.3 mm) was significantly greater than without any graft (1.1 mm). No differences were found for the clinical parameters like PPD, BOP, suppuration, or recession [5]. In another recent multi-center randomized controlled trial, reconstructive surgical therapy of peri-implantitis lesions with access flap vs. the additional use of DBBM with collagen was evaluated. The study results revealed that surgical peri-implantitis therapy leads to a mean PPD reduction of 3.7 mm, a radiographic defect fill of about 1 mm, and a reduction of the mean BOP in both groups without significant differences. The amount of recession and reduction of the width of the keratinized mucosa was less pronounced in the test compared to the control group [4]. Comparing the results in the present study with a vertical bone regeneration of 3.1 to 3.5 mm using autogenous bone chips to the results of studies using DBBM with a radiographic defect fill of 1.0 to 2.3 mm, our outcome seems to be favorable.

In the present study, after the decontamination of the implant surface, the defect was grafted with a connective tissue graft which was fixed subperiosteally to augment the soft tissue thickness to reduce the recession and improve the peri-implant soft tissue esthetics.

The data for soft tissue management in addition to regenerative surgical peri-implantitis therapy are very limited. Simultaneous soft tissue grafting using autogenous connective tissue graft at the time of implant placement had a measurable protective effect in a clinical study on the reduction of BOP, PD and of mucositis and peri-implantitis prevalence [41]. In a 1- to 8-year follow-up study on immediate implant placement in the presence of initial mucogingival recession, the results indicated that the simultaneous connective tissue graft had an positive impact on the reduction of the recession, the KMW, the vertical buccal bone regeneration, the buccal bone thickness and the risk of marginal bone resorption [42].

In a narrative review on soft tissue management in surgical peri-implantitis therapy, it was concluded that it is suitable to combine a surgical per-implantitis therapy with connective tissue grafting to maintain or improve esthetic results. Moreover, the increase of the width and thickness of the keratinized mucosa and the decrease of frenula tension and mucosal activity should be achieved prior to surgical interventions [29].

Although these 3-year results of this new approach for regenerative peri-implantitis surgery are very promising, prospective randomized studies are needed to examine the impact of the new flap design, the bone graft material, the soft tissue grafting and surface decontamination on the peri-implant hard and soft tissue regeneration.

## Conclusion

Marginal bone levels and soft tissue results suggest proof of principle for the regeneration of severe peri-implant hard and soft tissue deficiencies by the LAPIDER treatment approach. With the use of this concept, implant surface decontamination and significant improvements of the peri-implant hard and soft tissue are simultaneously possible.

## Data Availability

The dataset used and analyzed during the current publication is available from the corresponding author on reasonable request.

## Abbreviations

BOP: Bleeding on probing
CB-CT: Cone beam computed tomography
DBBM: Demineralized bovine bone mineral
FGG: Free gingival graft
KMT: Thickness of the keratinized mucosa
KMW: Width of keratinzed mucosa
LAPIDER: Laser-assisted peri-implant defect regeneration
MBL: Marginal bone level
PES: Pink Esthetic Score
PPD: Peri-implant probing depths
